# An *In Vivo C. elegans* Model System for Screening EGFR-Inhibiting Anti-Cancer Drugs

**DOI:** 10.1371/journal.pone.0042441

**Published:** 2012-09-05

**Authors:** Young-Ki Bae, Jee Young Sung, Yong-Nyun Kim, Sunshin Kim, Kyeong Man Hong, Heung Tae Kim, Min Sung Choi, Jae Young Kwon, Jaegal Shim

**Affiliations:** 1 Comparative Biomedicine Research Branch, National Cancer Center, Ilsandong-gu, Goyang-si, Gyeonggi-do, Korea; 2 Pediatric Oncology Research Branch, National Cancer Center, Ilsandong-gu, Goyang-si, Gyeonggi-do, Korea; 3 New Experimental Therapeutics Branch, National Cancer Center, Ilsandong-gu, Goyang-si, Gyeonggi-do, Korea; 4 Cancer Cell and Molecular Biology Branch, National Cancer Center, Ilsandong-gu, Goyang-si, Gyeonggi-do, Korea; 5 Center for Lung Cancer, National Cancer Center, Ilsandong-gu, Goyang-si, Gyeonggi-do, Korea; 6 Department of Biological Sciences, Sungkyunkwan University, Suwon, Gyeonggi-do, Korea; Brown University, United States of America

## Abstract

The epidermal growth factor receptor (EGFR) is a well-established target for cancer treatment. EGFR tyrosine kinase (TK) inhibitors, such as gefinitib and erlotinib, have been developed as anti-cancer drugs. Although non-small cell lung carcinoma with an activating EGFR mutation, L858R, responds well to gefinitib and erlotinib, tumors with a doubly mutated EGFR, T790M-L858R, acquire resistance to these drugs. The *C. elegans* EGFR homolog LET-23 and its downstream signaling pathway have been studied extensively to provide insight into regulatory mechanisms conserved from *C. elegans* to humans. To develop an *in vivo* screening system for potential cancer drugs targeting specific EGFR mutants, we expressed three LET-23 chimeras in which the TK domain was replaced with either the human wild-type TK domain (LET-23::hEGFR-TK), a TK domain with the L858R mutation (LET-23::hEGFR-TK[L858R]), or a TK domain with the T790M-L858R mutations (LET-23::hEGFR-TK[T790M-L858R]) in *C. elegans* vulval cells using the *let-23* promoter. The wild-type hEGFR-TK chimeric protein rescued the *let-23* mutant phenotype, and the activating mutant hEGFR-TK chimeras induced a multivulva (Muv) phenotype in a wild-type *C. elegans* background. The anti-cancer drugs gefitinib and erlotinib suppressed the Muv phenotype in LET-23::hEGFR-TK[L858R]-expressing transgenic animals, but not in LET-23::hEGFR-TK[T790M-L858R] transgenic animals. As a pilot screen, 8,960 small chemicals were tested for Muv suppression, and AG1478 (an EGFR-TK inhibitor) and U0126 (a MEK inhibitor) were identified as potential inhibitors of EGFR-mediated biological function. In conclusion, transgenic *C. elegans* expressing chimeric LET-23::hEGFR-TK proteins are a model system that can be used in mutation-specific screens for new anti-cancer drugs.

## Introduction

Development of a high-throughput, low-cost *in vivo* screening system for small molecule anti-cancer reagents would ideally be able to overcome the major problems of conventional *in vitro* screening methods. Due to fast generation time, high progeny numbers, low cost, and well established genetic tools, the nematode *Caenorhabditis elegans* (*C. elegans*) is an attractive candidate for an animal model screening system, with many of the advantages of *in vitro* screening systems and animal models [1].

EGFR is overexpressed or aberrantly activated in various types of human cancer, such as breast, ovarian, and non-small-cell lung carcinoma (NSCLC) [2]. EGFR is involved in various steps of cancer development including tumorigenesis, invasion, metastasis, and angiogenesis [3], and thus provides an attractive target for cancer drug development. Gefitinib (Commercial name: Iressa) was the first EGFR-TK inhibitor drug developed for the treatment of epithelial cancers such as NSCLC [4]. Mutations in the EGFR-TK domain have been linked to gefitinib sensitivity in a subset of lung cancers, and have also been found to activate anti-apoptotic pathways [5], [6].

*C. elegans* vulval development is a well-established model system used to study the EGFR signaling pathway [7]–[9]. Among the six vulval precursor cells (VPCs), P5.p, P6.p, and P7.p adopt the 2°-1°-2° cell fates, respectively, and continue dividing to form the mature vulva. The 1° cell fate is determined as a result of EGFR-Ras-MAPK signaling in P6.p, whereas the 2° cell fate is determined by LIN-12/Notch signaling in P5.p and P7.p, which is activated as a result of EGFR-Ras-MAPK signaling in the neighboring cell. Components of the EGFR pathway, including EGFR, Ras, Raf, MEK, and MAPK, are highly conserved between humans and *C. elegans* [8]. A limited number of chemical compounds that target the EGFR pathway have been tested using *C. elegans* vulval development as a model. Farnesyltransferase inhibitors, which inhibit Ras activity, and MCP compounds, which disrupt Ras-Raf interactions were found to act specifically on the orthologous proteins in the *C. elegans* EGFR-Ras pathway [10]–[12]. The toxicity of the EGFR kinase inhibitors BIBU1361 and BIBX1382 was also evaluated in *C. elegans* [13]. These studies suggest the potential for using *C. elegans* as a tool for anti-EGFR pathway drug screening.

In this study, we developed and analyzed a human EGFR-driven *C. elegans* model, which exhibits the Muv phenotype. Using this model, a pilot screen of 8,960 chemicals was conducted, and an EGFR inhibitor and a MEK inhibitor were isolated as suppressors, suggesting that this *C. elegans*-based system can be used efficiently to screen for new EGFR-inhibitory drugs.

## Materials and Methods

### Worm culture and strains

Wild-type N2 and mutant strains were cultured as described by Brenner [14]. Mutant alleles used in this work are *let-23(sy1), let-23(sa62), let-60(n1700)* and *lin-15(n765)*. Integration lines used in this work are *jgIs6*[*let-23p*::LET-23::hEGFR-TK[L858R], *rol-6(su1006)*], *jgIs19*[*let-23p*::LET-23::hEGFR, *rol-6(su1006)*], *jgIs25*[*let-23p*::LET-23::hEGFR-TK[T790M-L858R], *rol-6(su1006), myo-2p::mCherry*], *jgIs14*[*egl-17p*::EMR-1::RFP, *dhs-31p*::NLS::GFP, *rol-6(su1006)*], JJ1136 (HMP-1::GFP), PS4657 (AJM-1::GFP) and PS3352 (CDH-3::GFP).

### Construction of LET-23::hEGFR chimeric plasmids

We used genomic DNA of the *let-23* gene and cDNA encoding human EGFR. Each DNA fragment was amplified by PCR, cloned into the pGEM-T easy vector (Promega Inc., Madison, WI, USA), and confirmed by sequencing. We then assembled the DNA fragments using appropriate restriction enzymes and the corresponding sites of the pPD117.01 vector (Dr. Andrew Fire, Stanford Univ., CA, USA). QuikChange site-directed mutagenesis (Cat # 200523, Agilent Technologies Inc., Santa Clara, CA, USA) of EGFR-TK cDNA was performed to produce EGFR[L858R], EGFR[T790M] and EGFR[T790M-L858R]. The detailed procedure and primers used in this study are provided in the Supplementary Materials (Fig. S1B). To use as a secondary cell fate marker, pJG205 was constructed by combining a PCR fragment amplified from the genomic DNA sequence 4.0 kb upstream of *egl-17* with *emr-1* cDNA, DsRed (RFP, Clontech of TAKARA Bio Inc.) and pPD95.77 (A. Fire). The GFP encoding sequence of pPD95.77 was replaced with DsRed cDNA. Another cell fate marker, pJG207, was made by cloning the *dhs-31* promoter region into pPD95.69 (A. Fire), which contains the SV40 nuclear localization signal (NLS) and GFP. All plasmid constructs were confirmed by sequencing.

### Microinjection and integration

The injection concentration of DNA constructs and markers were 75 µg/ml pRF4[*rol-6(su1006)*], 50 or 25 µg/ml EGFR chimeric plasmids, 50 µg/ml *ttx-3::gfp*, 35 µg/ml pJG205[*egl-17p*::EMR-1::RFP], 40 µg/ml pJG207[*dhs-31p*::NLS::GFP], and 5 µg/ml pCFJ90[*myo-2p*::mCherry] (Addgene, Cambridge, MA, USA). The total DNA concentration of each injection mixture was 150 µg/ml, with pBluescript SK+ DNA added when necessary. All integrated lines were made using UV irradiation and out-crossed four times.

### Microscopy and Hoechst33342 staining

All microscopic images were captured and processed using an AxioCam HRc digital camera attached to an Imager M1 fluorescence microscope and Axiovision Rel. 4.6 software (Zeiss Inc., Germany). For Hoechst33342 (Molecular Probes of Life Technologies Co., Grand Island, NY, USA) staining, *jgIs6* worms were harvested and washed several times with M9 buffer. The worms were then soaked in 1 µg/ml Hoechest33342 solution for 30 minutes. After washing, worms were prepared for observation.

### Chemical treatment and statistics

Chemicals including gefitinib, erlotinib, U0126, AZD6244, PD0325901 and WZ4002 were purchased from Selleck Chemicals LLC. (Houston, TX, USA), and the 1,280 chemical library was purchased from Sigma-Aldrich Co. LLC. (St. Louis, MO, USA). The other 7,680 chemicals were obtained from the Korea Chemical Bank of KRICT. Chemicals were dissolved in 100% DMSO solution, and kept at −20°C as 1 or 10 mM stocks for screening. All chemical tests were executed in 96-well plates, and the final volume per well was 100 µl including worms, cholesterol, and dead *E. coli*. The DMSO concentration of the control group was kept at 0.5%, and DMSO concentrations of all experimental groups were below 0.5%. The final chemical concentration used for the screen was 5 µM and 20 to 50 L1 worms were cultured in each well of the 96-well plate. Each chemical test included at least three wells per chemical and was repeated at least twice. The error bars in all graphs represent SD (standard deviation), and *P* values relative to the control were calculated by unpaired Student's *t*-test.

## Results

### Chimeric LET-23::hEGFR-TK protein is functional in *C. elegans*

**Figure 1:**
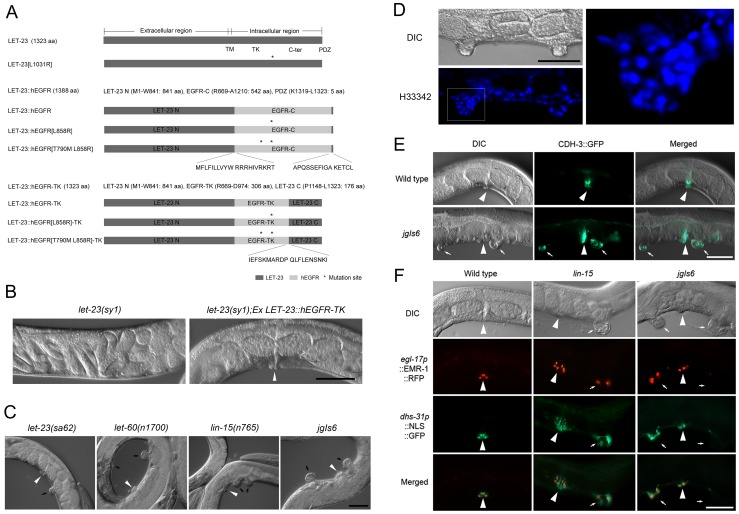
The development of the human oncogenic EGFR induced Muv model. (A) LET-23 and LET-23-based chimeric receptor constructs. All constructs were designed to express each chimeric receptor from the *let-23* promoter. (B) The LET-23::hEGFR-TK chimera rescues the vulvaless phenotype of the *let-23(sy1)* mutant. (C) A comparison of several Muv mutants and the *jgIs6* transgenic strain which expresses LET-23::hEGFR-TK[L858R]. *C. elegans* expressing LET-23::hEGFR-TK[L858R] exhibited a larger pseudovulva compared to *let-23(sa62)* and *let-60(n1700)* Muv mutants. (D) Hoechst33342 (H33342) staining of the pseudovulval region in *jgIs6* revealed many nuclei. The boxed region of the lower panel is enlarged in the right panel. (E) Expression of a 1° cell marker, CDH-3::GFP in the wild-type worm and *jgIs6*. CDH-3::GFP is highly expressed both in the vulva and pseudovulva of *jgIs6*. (F) Expression of 2° vulval cell fate markers in the *lin-15* Muv mutant and *jgIs6*. Reporter genes controlled by promoters of *egl-17* and *dhs-31* were expressed in the vulva and pseudovulva. Arrowheads indicate normal vulvae and small arrows indicate pseudovulvae. Scale bars, 50 µm.

To develop a *C. elegans* model system to screen for chemicals that inhibit human EGFR (hEGFR) activity, we designed plasmid constructs that express the *C. elegans* EGFR ortholog, LET-23, and the hEGFR fusion protein, by swapping the cytoplasmic or TK domain of LET-23 with each hEGFR domain (Fig. 1A and Fig. S1). We aimed to facilitate functional expression of the human counterpart in *C. elegans* by retaining most of the *C. elegans* LET-23 coding and regulatory sequences, for example, by maintaining C-terminal residues important for proper trafficking [15]. The putative protein products of these transgenes have 1388 amino acids (LET-23::hEGFR) or 1323 amino acids (LET-23::hEGFR-TK). As shown in Figure 1A and Figure S2, LET-23::hEGFR includes 841 amino acids of the LET-23 N-terminal domain, 542 amino acids of the human EGFR C-terminal domain, and 5 amino acids of the LET-23 PDZ interacting motif. LET-23::hEGFR-TK includes 841 amino acids of the LET-23 N-terminal domain, 306 amino acids of the human EGFR-TK domain, and 176 amino acids of the LET-23 C-terminal domain. To test whether this chimeric LET-23::hEGFR-TK protein is functional, the construct was microinjected into the *let-23(sy1)* mutant. Most *let-23* mutants are lethal, but *let-23(sy1)* is viable and vulvaless (Vul) due to aberrant trafficking of LET-23 [15]–[17]. The chimeric LET-23::hEGFR-TK protein rescued the Vul phenotype of *let-23(sy1)* (Fig. 1B), indicating that the chimeric protein is functional in *C. elegans*. When the *let-23(sy1)* mutant was rescued with *jgIs19*, an integrated strain containing the LET-23::hEGFR transgene, the *let-23(sy1);jgIs19* strain showed a significantly reduced vulvaless population (9.1%) compared to the *let-23(sy1)* mutant (92%) (Fig. S1C).

Next, we assessed the effects of over-expressing the activating mutant form of LET-23::hEGFR-TK in a *let-23*(+) background. Increased activity of the EGFR-Ras-MAPK pathway results in the hyper-induction of vulval cells, referred to as a Muv phenotype, as is seen with the semi-dominant *let-23(sa62)* mutation and the constitutively active *let-60(n1700)* mutation. *lin-15* acts upstream of *let-23* to negatively regulate the EGFR-Ras-MAPK pathway [18]. This negative regulation is disrupted in the *lin-15(n765)* mutant, resulting in a strong Muv phenotype [18], [19]. Thus, we expected that the activating mutations in the EGFR-TK region would also cause a Muv phenotype. Two activating EGFR mutations that confer gefitinib sensitivity to certain lung cancers were tested: EGFR[L858R] and EGFR[Δ747–752] [20], [21]. In-frame deletions in exon 19 including Δ747–749 (44%), and single point mutations in exon 21 including L858R (41%) are the most frequently found EGFR-TK activating mutations in NSCLC [20], . The gefitinib-resistant EGFR[T790M-L858R] mutation was also tested. T790M is a secondary mutation which endows gefitinib resistance to the L858R lesion [21]. Chimeric LET-23::hEGFR-TK containing any of these mutations induced the hyper-induction of vulval cells resulting in a Muv phenotype (Fig. 1C and Table 1). Transgenic animals expressing LET-23::hEGFR-TK[L858R] or LET-23::hEGFR-TK[T790M-L858R] had a phenotype similar to *lin-15(n765)*, with 2-4 large pseudovulvae. In contrast, transgenic animals expressing LET-23::hEGFR-TK[T790M] did not exhibit a Muv phenotype. To facilitate further analysis, we selected a LET-23::hEGFR-TK[L858R] transgenic line to generate *jgIs6*, a strain with the transgenic array integrated into the genome. Integration of the transgene greatly enhanced the penetrance of the Muv phenotype; 49% Muv before integration versus 94.6% Muv after integration (Table 1). Staining of nuclei with Hoechst33342 revealed the presence of many nuclei in the pseudovulval region (Fig. 1D), and this means that cell numbers were increased in the pseudovulva of our transgenic strain similar to other Muv mutants such as *let-60(n1700)*, and *lin-15(n765)*. Transgenic animals showed a rolling phenotype because pRF4[*rol-6(su1006)*] was used as a transgenic marker. To facilitate Muv phenotype detection, we introduced the *sqt-1(jg52)* mutation into the transgenic lines. This *sqt-1* mutant suppresses the rolling phenotype of *rol-6* without any obvious defects [23]. Unexpectedly, we found that the *sqt-1(jg52)* mutation enhanced the Muv phenotype of the EGFR transgenic lines (Table 2).

**Table 1:** The multivulva phenotype of transgenic strains expressing LET-23::hEGFR-TK proteins.

Name	% Muv^a^	Integration line (% Muv, *n*)
LET-23::hEGFR	0	*jgIs19* (0, 175)
LET-23::hEGFR[L858R]	78	
LET-23::hEGFR[T790M-L858R]	75	
LET-23::hEGFR-TK	0	
LET-23::hEGFR-TK[L858R]	49	*jgIs6* (94.6±1.33, 1465)
LET-23::hEGFR-TK[T790M]	0	
LET-23::hEGFR-TK[Δ747–752]	12	
LET-23::hEGFR-TK[T790M-L858R]	45	*jgIs25* (94.9±0.32, 410)
LET-23[L1031R]	10	

All transgenes were expressed by the *let-23* promoter, and transgenic lines which show highest stability were selected out of 2 or 3 stable lines for each transgene. 100 worms were counted for each transgenic line. The amino acid L1031 in LET-23 is analogous to L858 in EGFR.

a % Muv, the penetrance of Muv phenotype among transgenic animals.

**Table 2:** The *sqt-1* mutation enhances the Muv phenotype of integrated strains.

Strain	% Muv	Number
*jgIs6*	94.6±1.33	1465
*sqt-1(jg52);jgIs6*	97.4±0.42	2012
*jgIs25*	94.9±0.32	410
*sqt-1(jg52);jgIs25*	95.1±0.19	593
*jgIs26*	50.9±4.13	494
*sqt-1(jg52);jgIs26*	94.5±1.03	445

The *sqt-1(jg52)* mutation was introduced to suppress the Rol phenotype of the integrated strains. *sqt-1* effect is different in each integrated line. There was no difference between *jgIs25* and *sqt-1;jgIs25* (*P* = 0.834), but the Muv ratio of *jgIs6* is changed in the *sqt-1* mutant background (*P* = 0.00163). In particular, *jgIs26* which is another integration line of LET-23::hEGFR-TK[T790M-L858R] exhibited the dramatic increase of Muv in the *sqt-1* mutant background (*P*<0.001). This Rol suppression by *sqt-1* allowed us to score the multivulva more clearly compared to the rolling strain.

### The Muv phenotype of *jgIs6* is due to ectopic activation of the LET-23/EGFR pathway

To confirm that pseudovulvae formation in *jgIs6* is due to specific activation of the EGFR-Ras-MAPK pathway by the chimeric LET-23::hEGFR-TK[L858R] protein, we performed RNAi of genes in the EGFR-Ras-MAPK pathway. Consistent with ectopic activation of the EGFR-Ras-MAPK pathway, knock-down of genes downstream of *let-23*, including *let-60/Ras, mek-2/MEK*, and *mpk-1/MAPK*, suppressed the Muv phenotype of *jgIs6*. RNAi of the LET-23/EGFR upstream gene *lin-3/EGF* also suppressed the Muv phenotype. The Muv phenotype of another transgenic line, *jgIs25*, which expresses LET-23::hEGFR-TK[L858R-T790M], was also suppressed by RNAi of *lin-3, let-60, mek-2*, and *mpk-1*(Fig. S3A). Since the Wnt pathway acts in parallel to *lin-3* to maintain VPC competence [24], we also performed Wnt pathway gene knockdown by RNAi to test whether Wnt activity affects Muv formation in *jgIs25*. Two Wnt pathway genes (*bar-1* and *cwn-2*) were tested, because their knockdown phenotypes are associated with vulval development [25], [26]. Similar to *lin-3* knockdown, RNAi of *bar-1* or *cwn-2* resulted in suppression of the Muv phenotype of *jgIs25* (). We observed rare larval lethality from these RNAi experiments because synchronized L1 larvae were treated with RNAi, same as the same method we used for drug treatment described below. To confirm the lethal RNAi effect of EGFR downstream genes, we treated *jgIs25* L4 larvae with *let-60* or *mpk-1* RNAi, and counted the numbers of F1 progeny. Larval lethality was significantly increased by *let-60* or *mpk-1* RNAi (Fig. S3C).

We compared vulval cell fate markers in *jgIs6* and Muv mutants that have the EGFR-Ras-MAPK pathway activated. When the 1° cell fate marker was examined, *jgIs6* pseudovulvae showed expression of the 1° cell fate marker CDH-3::GFP (Fig. 1E). CDH-3 is a cadherin expressed in the 1° vul C, D, E, and F cells [27]. To test for 2° fate marker expression, we constructed an integrated transgenic line expressing both *egl-17p*::EMR-1::RFP and *dhs-31p*::NLS::GFP. EGL-17 is expressed in the 2° vul C and D cells, and DHS-31 in the 2° vul B1, B2, and D cells at the adult stage [27], [28]. EMR-1 is a homolog of the human integral nuclear membrane protein emerin [29], and therefore, causes localization to the nuclear envelope. In a wild-type background, *egl-17p*::EMR-1::RFP and *dhs-31p*::NLS::GFP are expressed at the nuclear envelope and nucleus of the 2° vulval cells, respectively. Both *lin-15(n765)* and *jgIs6* animals had similar patterns of expression of *egl-17p*::EMR-1::RFP and *dhs-31p*::NLS::GFP (Fig. 1F). For the further comparison of *jgIs6* and Muv mutants, we examined the expression of AJM-1::GFP and HMP-1::GFP, which are both expressed at the adherens junction and mark the boundaries of proliferating and differentiating epithelial cells [30]. AJM-1::GFP and HMP-1::GFP expression was observed in ventral invaginations, including putative pseudovulval regions in *jgIs6*, *let-60(n1700)*, and *lin-15(n765)* mutants at the L4 stage. These two junction markers were also observed in the pseudovulva of *jgIs6* at the adult stage (Fig. S4). Taken together, the results described above suggest that the *jgIs6* transgenic strain expressing the chimeric LET-23::hEGFR-TK[L858R] protein display characteristics that are consistent with over-activation of the EGFR pathway in the Muv mutants.

### Gefitinib and erlotinib inhibit the Muv phenotype of *jgIs6*

**Figure 2:**
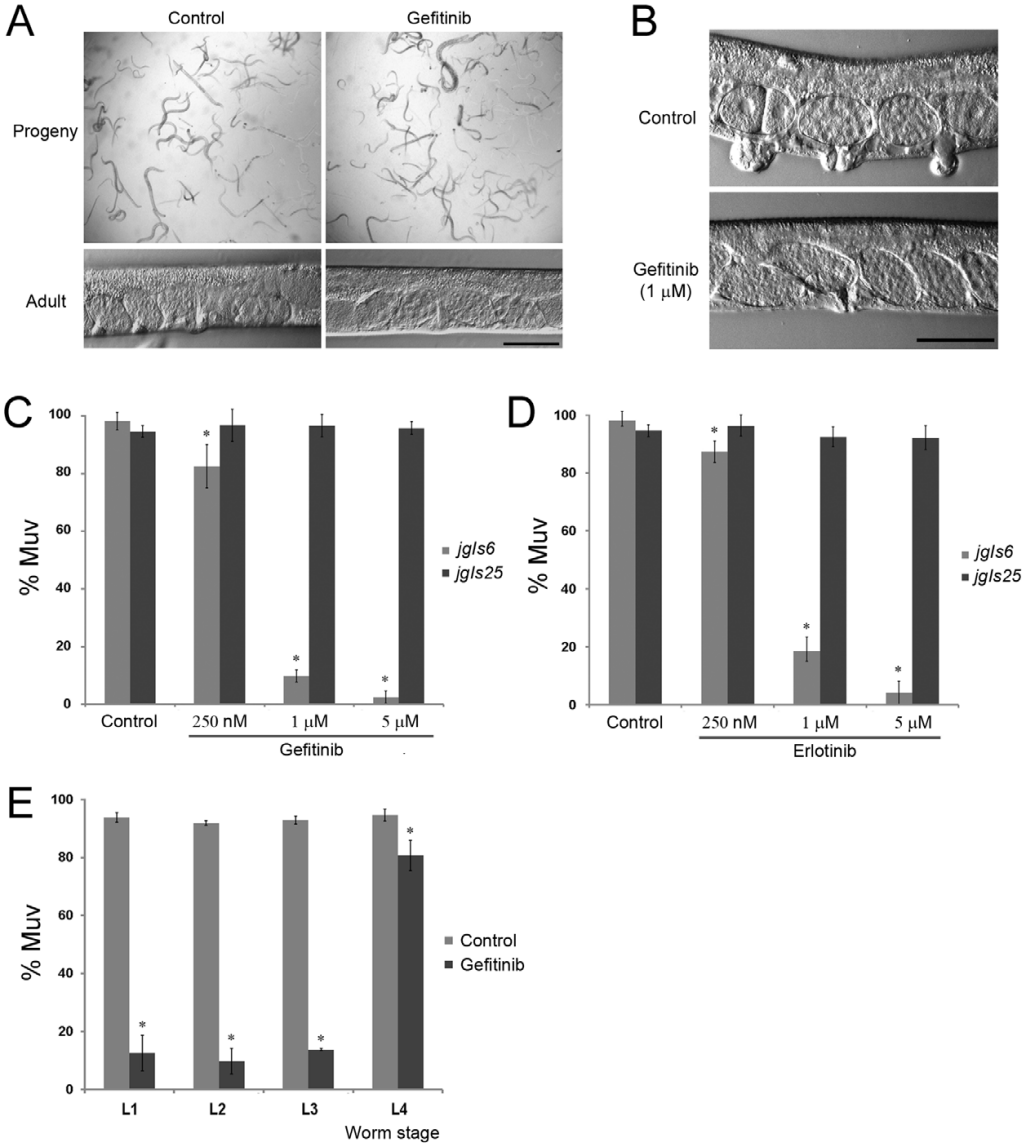
The Muv phenotype of *jgIs6* and *jgIs25* reflects similar responses as human cancers have against the anti-cancer drugs, gefitinib and elrotinib. (A) A high dose of gefitinib (40 µM) did not affect normal embryogenesis, larval growth, or vulval formation of the wild-type *C. elegans*. (B) Gefitinib inhibited the Muv phenotype of *jgIs6* which expresses LET-23::hEGFR-TK[L858R]. (C) Gefitinib inhibited the Muv phenotype of *jgIs6* in a dose dependent manner, but did not inhibit that of *jgIs25*, which expresses LET-23::hEGFR-TK[T790M-L858R]. Numbers of worms counted (*n*) were 159, 96, 130, 85, 87, 125, 108 and 88 from left along the X-axis. (D) Erlotinib produced a similar effect as gefitinib in both *jgIs6* and *jgIs25* models. (*n* = 159, 96, 67, 110, 80, 106, 95 and 176). (E) Determination of the developmental stage of *jgIs6* that is most responsive to gefitinib. As in normal vulval development, early larvae (L1–L3) responded well to gefitinib. (*n* = 128, 224, 134, 116, 139, 167, 99 and 66). Scale bars, 50 µm (A, B). X-axis, concentration of gefitinib (C), erlotinib (D) and worm stage of gefitinib treatment (E). Y-axis, % of worms showing the Muv phenotype (C–E). * *P*<0.001.

To determine whether the *jgIs6* transgenic strain expressing the chimeric LET-23::hEGFR-TK[L858R] protein could be used in a large-scale screen for human EGFR-TK inhibitors, we tested the effects of the drugs gefitinib and erlotinib. When wild-type *C. elegans* were treated with gefitinib, even high doses (40 µM) did not affect embryogenesis or larval development (Fig. 2A). This indicated that gefitinib is not toxic to wild-type *C. elegans*, and appears to have little to no effect on LET-23 function, which is not surprising given the low sequence identity with the human EGFR protein or correlation of gefitinib response and activating EGFR mutations [31]. In contrast, the Muv phenotype of *jgIs6* was inhibited up to 90% by 1 µM gefitinib and completely inhibited by 5 µM gefitinib (Fig. 2B and C). Considering that 250 or 500 mg of gefitinib orally once daily is recommended for cancer patients (approximately 10 µM) [4], [32], the inhibitory effect of gefitinib appears to be similar in *C. elegans* and humans. To verify the mechanism of action of reversible EGFR-TK inhibitors (TKIs) for our LET-23::hEGFR-TK-expressing transgenic *C. elegans*, we treated *jgIs25* with gefitinib. The T790M mutation may result in an alteration of EGFR topology that precludes the binding of reversible EGFR-TKIs through steric hindrance, or T790M may increase the affinity of the kinase domain for ATP [21], [33]–[35]. Gefitinib treatment did not inhibit the Muv phenotype of *jgIs25* (Fig. 2C). Erlotinib, another EGFR-TKI anti-cancer drug, produced similar inhibitory effects to gefitinib (Fig. 2D). We tried to compare the expression level of two chimeric proteins from *jgIs6* and *jgIs25*, but we were unable to quantify the chimeric protein. Assuming similar levels of transgenic expression, the observed difference in activity is likely due to a decrease in drug sensitivity conferred by the L858R mutation.

To determine the stage of worm development during which gefitinib treatment is most effective, we treated *jgIs6* of each larval stage with 1 µM gefitinib, and scored the Muv phenotype at the adult stage. Animals exposed to gefitinib from the L1, L2, or L3 stages showed a marked reduction in the Muv phenotype, and the degree of response was similar between the three stages (Fig. 2E). L4 animals were not as susceptible to gefitinib, indicating that gefitinib treatment before the L3/L4 molt, when vulval development initiates, is critical for effective inhibition of the LET-23::hEGFR-TK[L858R] protein. From this result, we concluded that early larvae, from L1 to L3, can be used for screening new inhibitors against the activated EGFR-TK signaling pathway.

### Pilot screen for inhibitors that suppress the Muv phenotype of *jgIs6*

**Figure 3:**
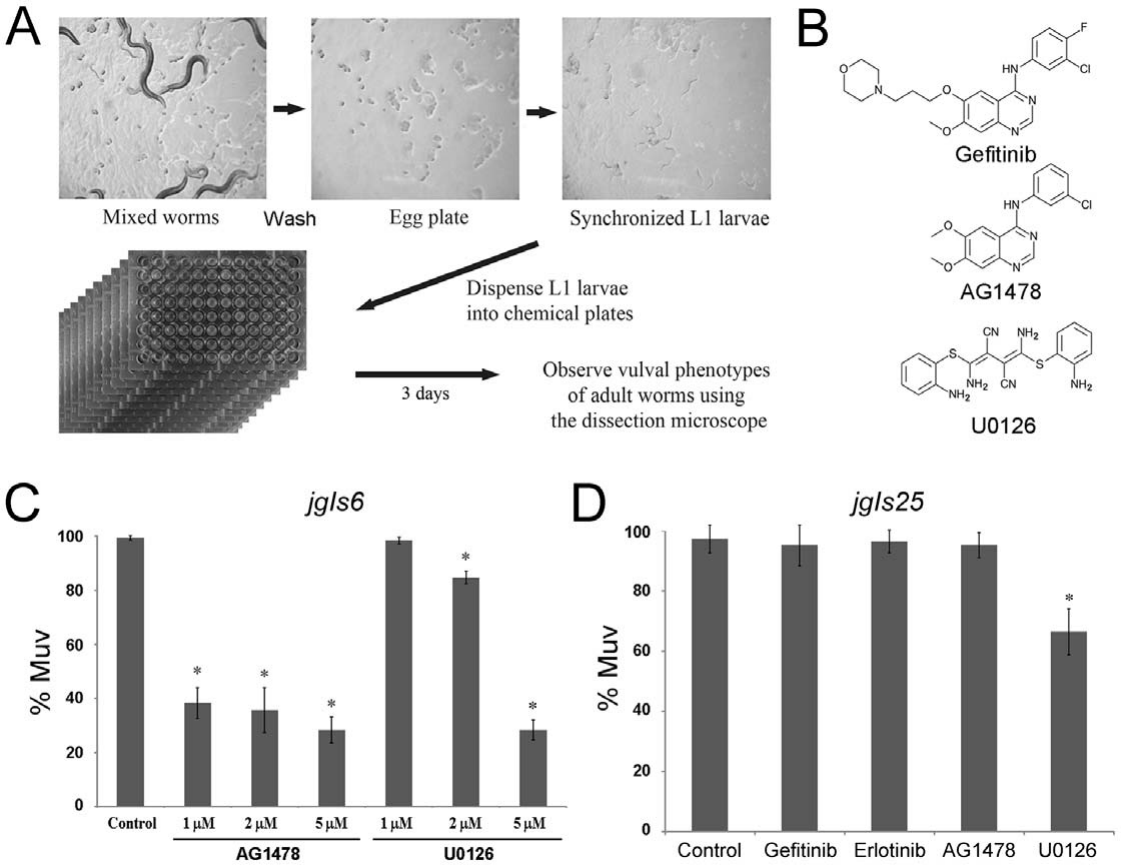
A pilot screen of 1,280 chemicals for EGFR-TK inhibitors using *jgIs6*. (A) Screening method, including synchronization of *C. elegans* and liquid culture using the 96-well plate for inhibitor screen. (B) Chemical structures of gefitinib, AG1478 and U0126. (C) Both AG1478 and U0126 inhibit the Muv phenotype of *jgIs6* in a dose dependent manner. (*n* = 295, 193, 381, 133, 254, 304 and 415 from left along the X-axis). (D) Effect of gefitinib, erlotinib, AG1478 and U0126 on *jgIs25*. Gefitinib, erlotinib, and AG1478 did not inhibit the Muv phenotype of *jgIs25*, but U0126 inhibited. (*n* = 81, 99, 106, 90 and 81). Chemical concentration, 5 µM. * *P*<0.001.

Based on our early results, we designed a protocol for large-scale high-throughput screening of EGFR inhibitors using *jgIs6*. To prepare a large number of synchronized larvae, *C. elegans* were grown until the plates were filled with eggs, and larvae and adults were removed by simple washing with M9 buffer. After 12 hours, hatched larvae were harvested and washed three times with M9 buffer. We dispensed L1 larvae into 96-well plates that contained a mix of dead *E. coli* and the chemicals being tested. After 3–4 days, the Muv phenotype was observed on a dissecting microscope (Fig. 3A). Using this protocol, we conducted a screen of 1,280 small molecules with known molecular targets and efficacy. Among the 1,280 chemicals, AG1478 and U0126 inhibited the Muv phenotype of *jgIs6*. AG1478 is an EGFR inhibitor frequently used in *in vitro* experiments, with a structure similar to gefitinib (Fig. 3B). U0126, which is known as an inhibitor of MEK, inhibited the *jgIs6* Muv phenotype at 5 µM, but not at lower concentrations (Fig. 3C). When the effects of AG1478 and U0126 on the gefitinib-resistant LET-23::hEGFR-TK[T790M-L858R] model *jgIs25* were tested, only U0126 had an inhibitory effect, whereas AG1478 was ineffective (Fig. 3D).

### MEK inhibitors may potentially treat gefitinib-resistant cancers

**Figure 4:**
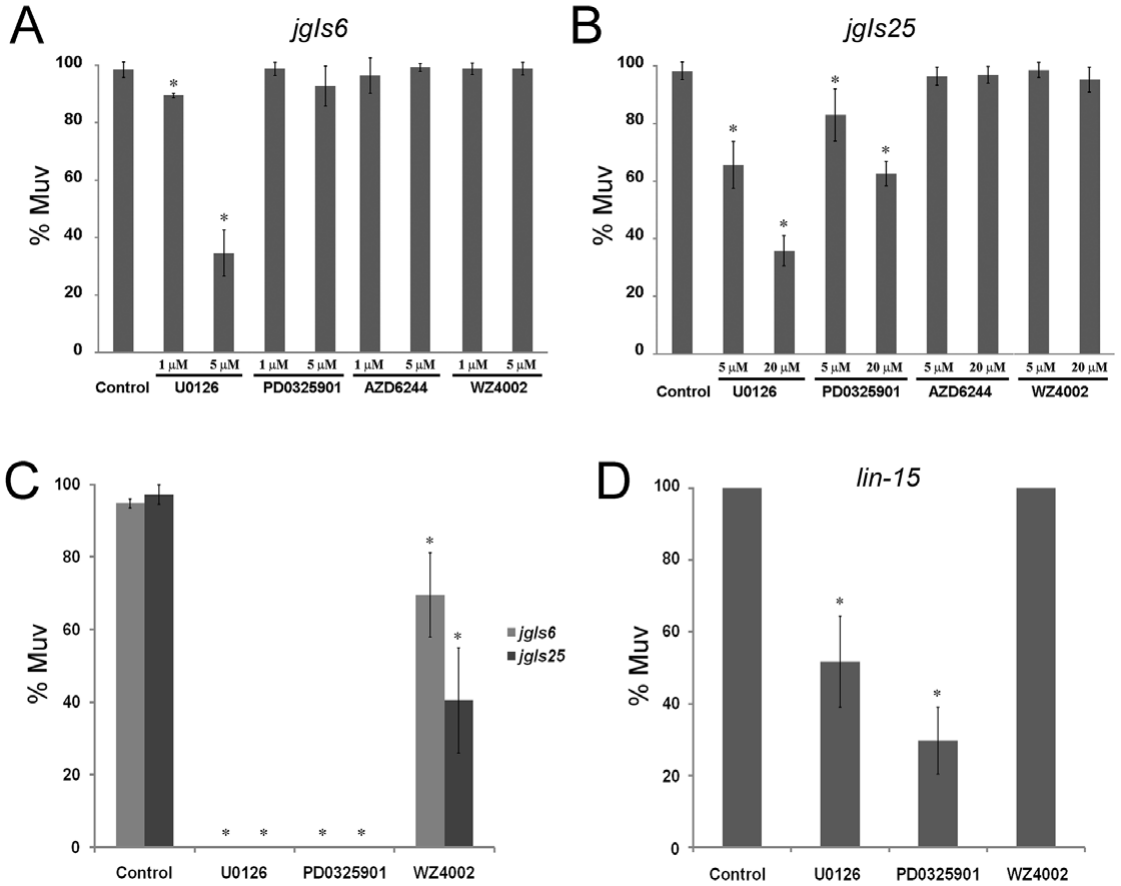
MEK inhibitors rescue the gefitinib-resistant Muv phenotype of *jgIs25*. (A) U0126, PD0325901 (MEK inhibitor), AZD6244 (RAF[V600E] inhibitor) and WZ4002 (EGFR[T790M] inhibitor) were added to *jgIs6*. (*n* = 86, 94, 116, 64, 88, 66, 79, 110 and 98 from left along the X-axis). (B) Chemicals were added to gefitinib resistant *jgIs25*. MEK inhibitors, U0126 and PD0325901, inhibited the Muv phenotype of *jgIs25*, but the other chemicals did not. (*n* = 64, 100, 128, 113, 89, 64, 63, 79 and 98). (C) Excessive doses (100 µM) of chemicals were added to *jgIs6* and *jgIs25*. Two MEK inhibitors perfectly inhibited the Muv phenotype of *jgIs6* and *jgIs25*. WZ4002 inhibited the Muv phenotype of *jgIs25* better than that of *jgIs6*. (*n* = 160, 213, 186, 312, 195, 323, 192 and 237). (D) The Muv phenotype of *lin-15* was suppressed by U0126 and PD0325901; chemical concentration, 100 µM (X-axis). (*n* = 119, 89, 90 and 143). * *P*<0.001.

The observation that U0126 inhibited the Muv phenotype of *jgIs25* suggested that some MEK inhibitors may have the potential to inhibit gefitinib-resistant forms of EGFR mutations. Therefore, we tested the effects of another MEK inhibitor, PD0325901, as well as a Raf[V600E] inhibitor (AZD6233), and an EGFR[T790M] inhibitor (WZ4002) in our model system. None of these chemicals inhibited the Muv phenotype of *jgIs6* animals at doses effective for U0126 (1 µM or 5 µM) (Fig. 4A). However, at higher doses (5 µM or 20 µM), the MEK inhibitor PD0325901 slightly inhibited the Muv phenotype of *jgIs25* (Fig. 4B). These results were confirmed in a side-by-side test, where *jgIs6* and *jgIs25* animals were treated with 100 µM of each chemical to observe the effects on the Muv phenotype. WZ4002, which is an inhibitor against the EGFR[T790M] gatekeeper mutation [36], inhibited the Muv phenotype of *jgIs25* better than that of *jgIs6*. Similar to U0126, PD0325901 perfectly inhibited the Muv phenotype of both *jgIs6* and *jgIs25* (Fig. 4C). We also tested whether these chemicals target *C. elegans* genes by treating the *lin-15* Muv mutant with U0126 and PD0325901. Both U0126 and PD0325901 suppressed the Muv phenotype of *lin-15* at an excessive concentration (Fig. 4D). This result suggests that *mek-2*, one of MEKs in *C. elegans* that is related to vulval development, is one of the possible target candidates of U0126 and PD0325901.

**Figure 5:**
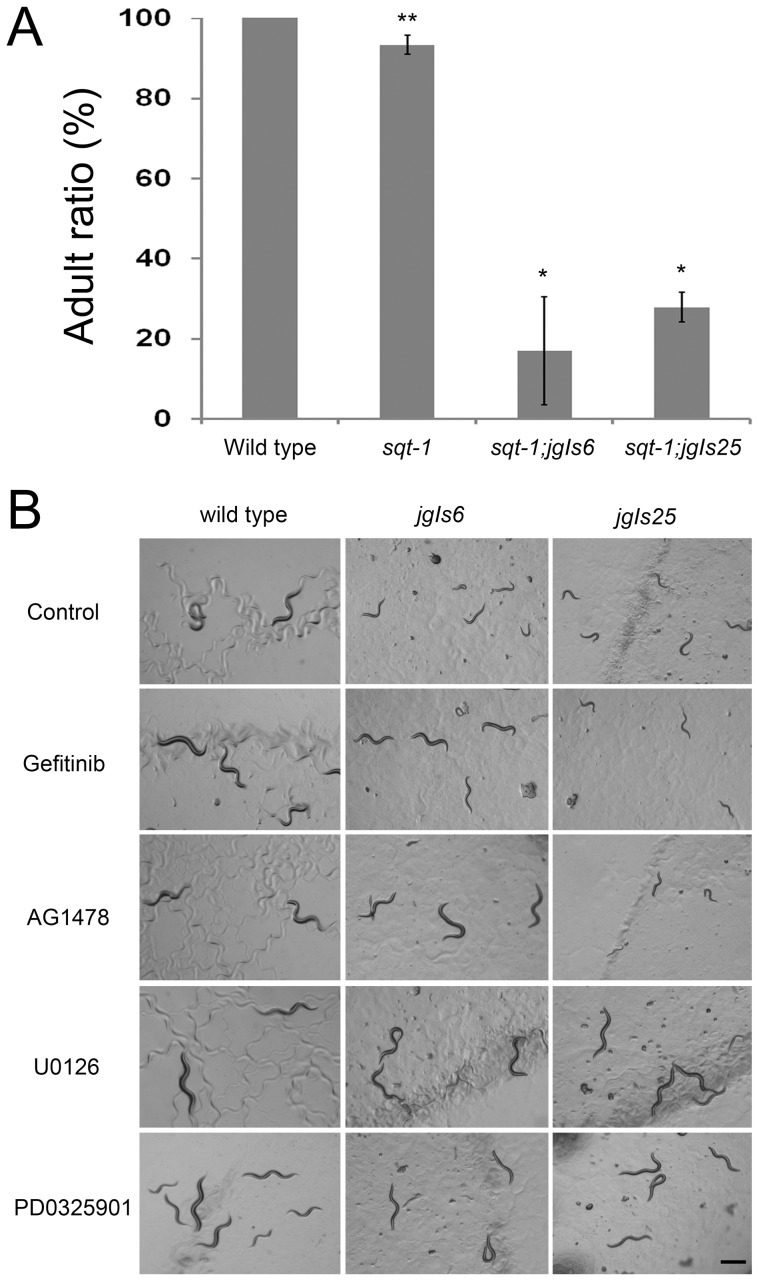
The Muv inhibitors suppressed the slow growth phenotype of *jgIs6* and *jgIs25*. (A) Ratios of adults 3 days after placing embryos on plate. All wild-type strains were adults, but most *jgIs6* and *jgIs25* transgenic strains were younger than the L4 stage. Five adults were transferred to each plate and were removed after 6 hours of egg-laying. Three days after removing P0 worms, F1 worms were observed. Four plates were counted for each strain. (*n* = 488, 313, 104 and 94 from left along the X-axis). * *P*<0.001 and ** *P*<0.05. (B) U0126 and PD0325901 suppressed the slow growth phenotype of *jgIs6* and *jgIs25*. Gefitinib and AG1478 suppressed the slow growth phenotype of *jgIs6*, but not *jgIs25*. L1 stage larvae were incubated with each chemical for 4 days in 96-well plates, and they were recovered on a fresh plate for 6 hours before taking pictures. *C. elegans* grew slowly when cultured in 96-well plates. Control (0.5% DMSO) and chemical concentration (50 µM). Scale bar, 500 µm.

All transgenic strains expressing the activated EGFR chimeras, including *jgIs6* and *jgIs25*, grow slowly (Fig. 5A and Fig. S5) similar to transgenic strains ectopically expressing LIN-3/EGF [37]. Interestingly, EGFR-TK inhibitors repaired the growth rate of *jgIs6* to the level of wild type, and U0126 and PD0325901 rescued the growth rate of both *jgIs6* and *jgIs25* (Fig. 5B).

## Discussion

We constructed transgenic *C. elegans* containing several different EGFR constructs. Transgenic lines expressing LET-23::hEGFR chimeric receptors exhibited a much stronger phenotype than those expressing LET-23::hEGFR-TK chimeric receptors (Table 1). Unfortunately, we failed to get integration lines for those constructs and only have data produced from the integration lines of LET-23::hEGFR-TK transgenic lines. The cytoplasmic tail of hEGFR may have evolved to transmit the activated signals more efficiently than LET-23, *C. elegans* EGFR, even in VPCs.

To establish our model system, the chimeric LET-23::hEGFR-TK transgene was expressed in a *let-23*(+) background. The potential formation of heterodimers of endogenous LET-23 with the chimeric LET-23::hEGFR-TK protein may explain some observations that were made over the course of our study. We occasionally observed that high doses of gefitinib inhibited normal vulval development in *jgIs6*. Also, RNAi of the EGFR upstream gene, *lin-3/EGF*, suppressed the Muv phenotype of *jgIs6* and *jgIs25* (Fig. S3A), which may be explained by heterodimerization of the endogenous and transgenic chimeric protein; although, the early role of *lin-3* in establishing and maintaining vulval cell competence may contribute to this phenotype [24]. In addition, Wnt pathway gene knockdown by RNAi affected the Muv formation in *jgIs25* (Fig. S3B). The Wnt pathway acts in parallel to *lin-3* during VPC competence [24]. As in many previous studies, vulval development in *jgIs6* and *jgIs25* appears to involve several signaling pathways rather than simple EGFR activation. Nevertheless, expressing the chimeric transgene in a *let-23*(+) background produces an advantage for screening purposes compared to a *let-23* null background. If the LET-23::EGFR-TK transgene were expressed in a *let-23* null mutant, inhibitor treatment would cause lethality or slow growth, making it difficult to distinguish true inhibitors of human EGFR-TK from chemicals that inhibit other endogenous essential genes or chemicals that display toxicity.

The chimeric LET-23::hEGFR-TK model system was designed for screening inhibitors that target the tyrosine kinase domain of human EGFR. As expected, well-known EGFR inhibitors, such as gefitinib or erlotinib which target the TK domain, were effective in suppressing the Muv phenotype in our model, but have little to no effect on the wild-type *C. elegans*. In addition, the chemicals that inhibit the Muv phenotype of *jgIs6* in another screen of 7,680 chemicals were similar in structure to gefitinib (Fig. S6). Thus, this approach has an advantage in screening for chemicals targeting specific protein domains.

This model system can be easily modified to screen for new chemicals that target drug-resistant cancers. Gefitinib resistance is caused by secondary mutations in EGFR [21], over-expression of c-Met [38] or IGFR [39], or mutations in EGFR downstream genes, such as *Ras* [40]. Here, we identified potential inhibitors of gefitinib-resistant secondary EGFR mutations. Gefitinib effectively suppressed the Muv phenotype of transgenic strains expressing EGFR mutations such as EGFR[L858R] (*jgIs6*), and was ineffective on gefitinib-resistant mutations, such as EGFR[T790M-L858R] (*jgIs25*). Using this model system, a MEK inhibitor was identified as a potential inhibitor of gefitinib-resistant EGFR mutations. In our models, MEK inhibitors produced a much stronger effect than WZ4002, which targets the EGFR[T790M] mutation [36] (Fig. 4C). Similarly, one could use transgenic *C. elegans* lines simultaneously expressing oncogenic EGFR and c-Met for drug screening, or use transgenic lines expressing chimeras of *C. elegans* and human EGFR downstream genes such as *Raf* or *MEK* [20], [41].

Because we screened inhibitors by observing the Muv phenotype using the dissection microscope, high-throughput screening (HTS) is difficult at a small laboratory level. However, *C. elegans* is a versatile model system, easily adapted for HTS. Chemical screening methods using *C. elegans* in automated bio-sorting machines, such as COPAS (Complex Object Parametric Analyzer and Sorter) have been reported [42], [43]. The use of fluorescence markers in conjunction with COPAS, such as AJM-1::GFP or *egl-17p*::EMR-1::RFP to mark the pseudovulvae, could enable high-throughput screening methods for new anti-cancer drugs in our model system. We propose another screening method that uses *jgIs6* or *jgIs25* would perform better than the one used in this study. These transgenic strains grow slowly and EGFR pathway inhibitors suppress the growth phenotype (Fig. 5). With these strains, we will be able to screen inhibitors first by selecting fast growing *C. elegans* and then confirm the Muv phenotype suppression.

By expressing a chimera of the *C. elegans* and human EGFR proteins in *C. elegans*, we have developed and characterized an animal model system that can be used to screen EGFR inhibitor anti-cancer drugs. The transgenic *C. elegans* have a multivulva phenotype, which is consistent with the activated EGFR pathway. In a pilot screen of 8,960 molecules, chemicals such as AG1478, which share a common backbone structure with gefitinib and erlotinib, as well as a MEK inhibitor, U0126, inhibited the Muv phenotype of *jgIs6*. We also provide evidence that MEK inhibitors may be effective in treating cancers that are resistant to known EGFR-TKIs. The humanized *C. elegans* makes a highly specific *in vivo* animal screening model system.

## Supporting Information

Figure S1 **Plasmid constructs for expressing LET-23::hEGFR chimeric receptors.**
(PDF)

Figure S2 **Amino acid sequences of the LET-23::hEGFR-TK and LET-23::hEGFR transgene products.**
(PDF)

Figure S3 **Knock-down of Ras-MAPK and Wnt pathway genes in** ***jgIs6*** **and** ***jgIs25*** **by feeding RNAi.**
(PDF)

Figure S4 **Expression of epithelial junction proteins in the** ***jgIs6*** **transgenic worm which expresses LET-23::hEGFR-TK[L858R].**
(PDF)

Figure S5 **Adult ratios of wild type and two integrated strains over time.**
(PDF)

Figure S6 **Seven chemicals found to inhibit the Muv phenotype of** ***jgIs6*** **in another screen are similar in structure to gefitinib.**
(PDF)

## References

[1] Artal-Sanz M, de Jong L, Tavernarakis N (2006) *Caenorhabditis elegans*: a versatile platform for drug discovery. Biotechnol J 1: 1405–1418. 17109493 10.1002/biot.200600176

[2] Hynes NE, Lane HA (2005) ERBB receptors and cancer: the complexity of targeted inhibitors. Nat Rev Cancer 5: 341–354. 15864276 10.1038/nrc1609

[3] Gschwind A, Fischer OM, Ullrich A (2004) The discovery of receptor tyrosine kinases: targets for cancer therapy. Nat Rev Cancer 4: 361–370. 15122207 10.1038/nrc1360

[4] Muhsin M, Graham J, Kirkpatrick P (2003) Gefitinib. Nat Rev Drug Discov 2: 515–516. 12841190 10.1038/nrd1136

[5] Pao W, Miller V, Zakowski M, Doherty J, Politi K, et al (2004) EGF receptor gene mutations are common in lung cancers from “never smokers” and are associated with sensitivity of tumors to gefitinib and erlotinib. Proc Natl Acad Sci U S A 101: 13306–13311. 15329413 10.1073/pnas.0405220101 PMC516528

[6] Sordella R, Bell DW, Haber DA, Settleman J (2004) Gefitinib-sensitizing EGFR mutations in lung cancer activate anti-apoptotic pathways. Science 305: 1163–1167. 15284455 10.1126/science.1101637

[7] Sternberg PW (2005) Vulval development. WormBook 1–28. 10.1895/wormbook.1.6.1 PMC4781130 18050418

[8] Chang C, Sternberg PW (1999) *C. elegans* vulval development as a model system to study the cancer biology of EGFR signaling. Cancer Metastasis Rev 18: 203–213. 10728984 10.1023/a:1006317206443

[9] Moghal N, Sternberg PW (2003) The epidermal growth factor system in *Caenorhabditis elegans* . Exp Cell Res 284: 150–159. 12648474 10.1016/s0014-4827(02)00097-6

[10] Hara M, Han M (1995) Ras farnesyltransferase inhibitors suppress the phenotype resulting from an activated *ras* mutation in *Caenorhabditis elegans* . Proc Natl Acad Sci U S A 92: 3333–3337. 7536929 10.1073/pnas.92.8.3333 PMC42160

[11] Gonzalez-Perez V, Reiner DJ, Alan JK, Mitchell C, Edwards LJ, et al (2010) Genetic and functional characterization of putative Ras/Raf interaction inhibitors in *C. elegans* and mammalian cells. J Mol Signal 5: 2. 20178605 10.1186/1750-2187-5-2 PMC2848644

[12] Lackner MR, Kindt RM, Carroll PM, Brown K, Cancilla MR, et al (2005) Chemical genetics identifies Rab geranylgeranyl transferase as an apoptotic target of farnesyl transferase inhibitors. Cancer Cell 7: 325–336. 15837622 10.1016/j.ccr.2005.03.024

[13] Dengg M, van Meel JC (2004) *Caenorhabditis elegans* as model system for rapid toxicity assessment of pharmaceutical compounds. J Pharmacol Toxicol Methods 50: 209–214. 15519907 10.1016/j.vascn.2004.04.002

[14] Brenner S (1974) The genetics of *Caenorhabditis elegans* . Genetics 77: 71–94. 4366476 10.1093/genetics/77.1.71 PMC1213120

[15] Kaech SM, Whitfield CW, Kim SK (1998) The LIN-2/LIN-7/LIN-10 complex mediates basolateral membrane localization of the *C. elegans* EGF receptor LET-23 in vulval epithelial cells. Cell 94: 761–771. 9753323 10.1016/s0092-8674(00)81735-3 PMC3224769

[16] Aroian RV, Lesa GM, Sternberg PW (1994) Mutations in the *Caenorhabditis elegans let-23* EGFR-like gene define elements important for cell-type specificity and function. EMBO J 13: 360–366. 8313880 10.1002/j.1460-2075.1994.tb06269.x PMC394816

[17] Aroian RV, Koga M, Mendel JE, Ohshima Y, Sternberg PW (1990) The *let-23* gene necessary for *Caenorhabditis elegans* vulval induction encodes a tyrosine kinase of the EGF receptor subfamily. Nature 348: 693–699. 1979659 10.1038/348693a0

[18] Huang LS, Tzou P, Sternberg PW (1994) The *lin-15* locus encodes two negative regulators of *Caenorhabditis elegans* vulval development. Mol Biol Cell 5: 395–411. 8054684 10.1091/mbc.5.4.395 PMC301050

[19] Clark SG, Lu X, Horvitz HR (1994) The *Caenorhabditis elegans* locus *lin-15*, a negative regulator of a tyrosine kinase signaling pathway, encodes two different proteins. Genetics 137: 987–997. 7982579 10.1093/genetics/137.4.987 PMC1206075

[20] Sharma SV, Bell DW, Settleman J, Haber DA (2007) Epidermal growth factor receptor mutations in lung cancer. Nat Rev Cancer 7: 169–181. 17318210 10.1038/nrc2088

[21] Kobayashi S, Boggon TJ, Dayaram T, Janne PA, Kocher O, et al (2005) EGFR mutation and resistance of non-small-cell lung cancer to gefitinib. N Engl J Med 352: 786–792. 15728811 10.1056/NEJMoa044238

[22] Gazdar AF (2009) Activating and resistance mutations of EGFR in non-small-cell lung cancer: role in clinical response to EGFR tyrosine kinase inhibitors. Oncogene 28 Suppl 1: S24–31. 19680293 10.1038/onc.2009.198 PMC2849651

[23] Kim TH, Kim YJ, Cho JW, Shim J (2011) A novel zinc-carboxypeptidase SURO-1 regulates cuticle formation and body morphogenesis in *Caenorhabditis elegans* . FEBS Lett 585: 121–127. 21094156 10.1016/j.febslet.2010.11.020

[24] Myers TR, Greenwald I (2007) Wnt signal from multiple tissues and *lin-3*/EGF signal from the gonad maintain vulval precursor cell competence in *Caenorhabditis elegans* . Proc Natl Acad Sci U S A 104: 20368–20373. 18077322 10.1073/pnas.0709989104 PMC2154437

[25] Green JL, Inoue T, Sternberg PW (2007) The *C. elegans* ROR receptor tyrosine kinase, CAM-1, non-autonomously inhibits the Wnt pathway. Development 134: 4053–4062. 17942487 10.1242/dev.005363

[26] Inoue T, Oz HS, Wiland D, Gharib S, Deshpande R, et al (2004) *C. elegans* LIN-18 is a Ryk ortholog and functions in parallel to LIN-17/Frizzled in Wnt signaling. Cell 118: 795–806. 15369677 10.1016/j.cell.2004.09.001

[27] Inoue T, Wang M, Ririe TO, Fernandes JS, Sternberg PW (2005) Transcriptional network underlying *Caenorhabditis elegans* vulval development. Proc Natl Acad Sci U S A 102: 4972–4977. 15749820 10.1073/pnas.0408122102 PMC555976

[28] Burdine RD, Chen EB, Kwok SF, Stern MJ (1997) *egl-17* encodes an invertebrate fibroblast growth factor family member required specifically for sex myoblast migration in *Caenorhabditis elegans* . Proc Natl Acad Sci U S A 94: 2433–2437. 9122212 10.1073/pnas.94.6.2433 PMC20105

[29] Lee KK, Gruenbaum Y, Spann P, Liu J, Wilson KL (2000) *C. elegans* nuclear envelope proteins emerin, MAN1, lamin, and nucleoporins reveal unique timing of nuclear envelope breakdown during mitosis. Mol Biol Cell 11: 3089–3099. 10982402 10.1091/mbc.11.9.3089 PMC14977

[30] Labouesse M (2006) Epithelial junctions and attachments. WormBook 1–21. 10.1895/wormbook.1.56.1 PMC4781617 18050482

[31] Paez JG, Janne PA, Lee JC, Tracy S, Greulich H, et al (2004) EGFR mutations in lung cancer: correlation with clinical response to gefitinib therapy. Science 304: 1497–1500. 15118125 10.1126/science.1099314

[32] Herbst RS, Fukuoka M, Baselga J (2004) Gefitinib- a novel targeted approach to treating cancer. Nat Rev Cancer 4: 956–965. 15573117 10.1038/nrc1506

[33] Kwak EL, Sordella R, Bell DW, Godin-Heymann N, Okimoto RA, et al (2005) Irreversible inhibitors of the EGF receptor may circumvent acquired resistance to gefitinib. Proc Natl Acad Sci U S A 102: 7665–7670. 15897464 10.1073/pnas.0502860102 PMC1129023

[34] Pao W, Miller VA, Politi KA, Riely GJ, Somwar R, et al (2005) Acquired resistance of lung adenocarcinomas to gefitinib or erlotinib is associated with a second mutation in the EGFR kinase domain. PLoS Med 2: e73. 15737014 10.1371/journal.pmed.0020073 PMC549606

[35] Yun CH, Mengwasser KE, Toms AV, Woo MS, Greulich H, et al (2008) The T790M mutation in EGFR kinase causes drug resistance by increasing the affinity for ATP. Proc Natl Acad Sci U S A 105: 2070–2075. 18227510 10.1073/pnas.0709662105 PMC2538882

[36] Zhou W, Ercan D, Chen L, Yun CH, Li D, et al (2009) Novel mutant-selective EGFR kinase inhibitors against EGFR T790M. Nature 462: 1070–1074. 20033049 10.1038/nature08622 PMC2879581

[37] Van Buskirk C, Sternberg PW (2007) Epidermal growth factor signaling induces behavioral quiescence in *Caenorhabditis elegans* . Nat Neurosci 10: 1300–1307. 17891142 10.1038/nn1981

[38] Engelman JA, Zejnullahu K, Mitsudomi T, Song Y, Hyland C, et al (2007) MET amplification leads to gefitinib resistance in lung cancer by activating ERBB3 signaling. Science 316: 1039–1043. 17463250 10.1126/science.1141478

[39] Cappuzzo F, Toschi L, Tallini G, Ceresoli GL, Domenichini I, et al (2006) Insulin-like growth factor receptor 1 (IGFR-1) is significantly associated with longer survival in non-small-cell lung cancer patients treated with gefitinib. Ann Oncol 17: 1120–1127. 16600976 10.1093/annonc/mdl077

[40] Uchida A, Hirano S, Kitao H, Ogino A, Rai K, et al (2007) Activation of downstream epidermal growth factor receptor (EGFR) signaling provides gefitinib-resistance in cells carrying EGFR mutation. Cancer Sci 98: 357–363. 17270025 10.1111/j.1349-7006.2007.00387.x PMC11160083

[41] Wong KK (2009) Recent developments in anti-cancer agents targeting the Ras/Raf/MEK/ERK pathway. Recent Pat Anticancer Drug Discov 4: 28–35. 19149686 10.2174/157489209787002461

[42] Sprando RL, Olejnik N, Cinar HN, Ferguson M (2009) A method to rank order water soluble compounds according to their toxicity using *Caenorhabditis elegans*, a Complex Object Parametric Analyzer and Sorter, and axenic liquid media. Food Chem Toxicol 47: 722–728. 19162123 10.1016/j.fct.2009.01.007

[43] Burns AR, Kwok TC, Howard A, Houston E, Johanson K, et al (2006) High-throughput screening of small molecules for bioactivity and target identification in *Caenorhabditis elegans* . Nat Protoc 1: 1906–1914. 17487175 10.1038/nprot.2006.283

