# Supplementary material for: An In Vivo C. elegans Model System for Screening EGFR-Inhibiting Anti-Cancer Drugs
Source: PLoS One. 2012 Sep 5;7(9):e42441. doi: 10.1371/journal.pone.0042441 (PMC3434183; doi:10.1371/journal.pone.0042441)
Supplement: Figure S1 — Plasmid constructs for expressing LET-23::hEGFR chimeric receptors. (PDF) [file pone.0042441.s001.pdf]

A

```
                                                               *
LET-23   859  PELT-PIDASVR-PNMSRICLIPSSELQTKLDKKLGAGAFGTVFAGIYYP  906
EGFR     686  RELVEPLTPSGEAPNQALLRILKETEF--KKIKVLGSGAFGTVYKGLWIP  733

LET-23   907  KRAKNVKIPVAIKVFQ--TDQSQTDEMLEEATNMFRLRHDNLLKIIGFCM  954
EGFR     734  EGEK-VKIPVAIKELREATSPKANKEILDEAYVMASVDNPHVCRLLGICL  782
                             Δ747-752
                      *
LET-23   955  HDDGLKIVTIYRPLGNLQNFLKLHKENLGAREQVLYCYQIASGMQYLEKQ  1004
EGFR     783  TST-VQLITQLMPFGCLLDYVREHKDNIGSQYLLNWCVQIAKGMNYLEDR  832
                      T790M                      *
LET-23  1005  VVHRDLATRNVLVKKFNHVEITDFGLSKILKHDADSITIKSGKVAIKWLA  1054
EGFR     833  LVHRDLAARNVLVKTPQHVKITDFGLAKLLGAEEKEYHAEGGKVPIKWMA  882
                                         L858R
LET-23  1055  IEIFSKHCYTHASDVWAFGVTCWEIITFGQSPYQGMSTDSIHNFLKDGNR  1104
EGFR     883  LESILHRIYTHQSDVWSYGVTVWELMTFGSKPYDGIPASEISSILEKGER  932

LET-23  1105  LSQPPNCSQDLYQELLRCWMADPKSRP  1132
EGFR     933  LPQPPICTIDVYMIMVKCWMIDADSRP  959
```

B

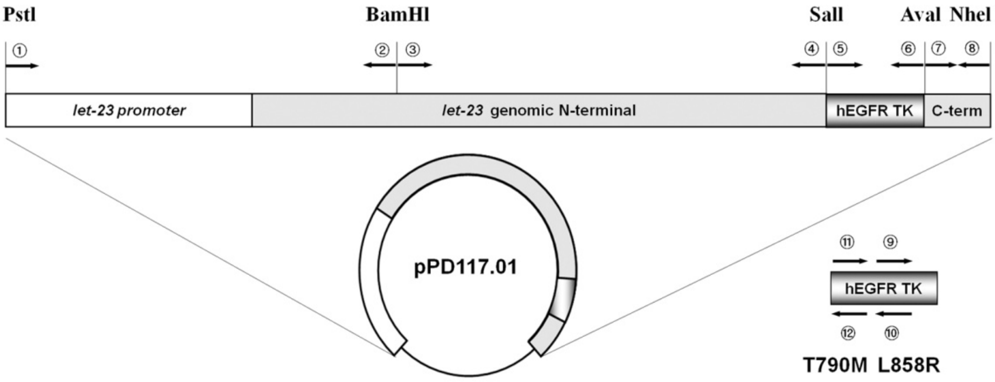

Primers	Sequences
① ZK1067.1-8 (Let23-pro-PstI-5′)	5′ TACTGCAGAATGAAACATCAAATGTATGG 3′
② ZK1067.1-9 (Let23-Exo5-BamHI-3′)	5′ TCAAGGATCCCCAACATTTATCATTAC 3′
③ ZK1067.1-10 (Let23-Exo5-BamH-5′)	5′ TCAAGGATCCCCAACATTTATCATTAC 3′
④ ZK1067.1-11 (Let23-TM-SalI-3′)	5′ TTGTCGACGCCAGTAGACGAGCTGTAAATTTC 3′
⑤ hEGFR-12 (hEGFR-TK-SalI-5′)	5′ ATCGTCGACGCCACATCGTTCGGAAGC 3′
⑥ hEGFR-24 (hEGFR-TK-AvaI-3′)	5′ GTCTCGGGCCATTTTGGAGAATTCGATG 3′
⑦ ZK1067.1-12 (Let23-TK-C 5′)	5′ ACCCGAG ACCCCCAACTTTTCCTGGAAAATTC 3′
⑧ ZK1067.1-13 (Let23-TK-C 3′)	5′ TTGCTAGCTTAAAGACAAGTTTCCTTTTGTG 3′
⑨ hEGFR-16 (L858R-5′)	5′ GATCACAGATTTTGGGCGGGCCAAACTGCTGGG 3′
⑩ hEGFR-17 (L858R-3′)	5′ CCCAGCAGTTTGGCCCGCCCAAAATCTGTGATC 3′
⑪ hEGFR-20 (T790M-5′)	5′ CCACCGTGCAGCTCATCATGCAGCTCATGCCCTTCG 3′
⑫ hEGFR-21 (T790M-3′)	5′ CGAAGGGCATGAGCTGCATGATGAGCTGCACGGTGG 3′

C

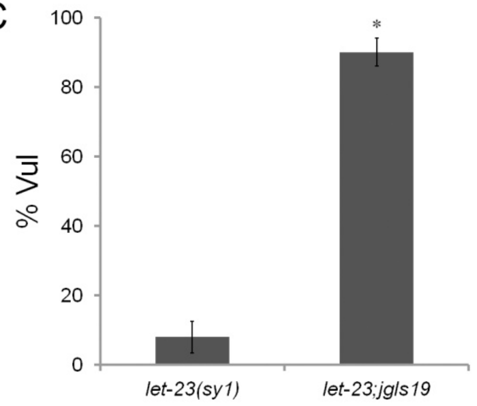

Figure S1. Plasmid constructs for expressing LET-23::hEGFR chimeric receptors. (A) Sequence alignment of tyrosine kinase domains of LET-23 and human EGFR. TK domains of LET-23 and EGFR exhibit relatively high conservation. Mutation sites found in lung cancer patients are also conserved. Shaded amino acids are positive and bolded amino acids are identical between two proteins. * The mutation sites of the activating EGFR variants. (B) Primers used for constructing LET-23::hEGFR chimeric receptors. All plasmids have the 4 kb *let-23* promoter region, LET-23 extracellular/transmembrane region (LET-23 N-terminal domain), human EGFR tyrosine kinase domain (EGFR-TK) and LET-23 C-terminal domain. The difference between the wild-type and mutant EGFR chimeras is that mutant EGFR constructs have mutations in EGFR-TK domain. Arrows and numbers indicate orientation and sequence of primers used in this cloning. *Pst*I, *BamH*I, *Sal*I, *Ava*I and *Nhe*I indicate restriction enzyme sites used in practice. T790M and L858R mutations were introduced by site-directed mutagenesis. (C) *let-23(sy1)* rescue with *jgIs19*, an integrated strain expressing LET-23::hEGFR. The vulvaless population is reduced significantly in *let-23(sy1);jgIs19* (9.1±4.02 %, *n*=175) compared to *let-23(sy1)* (92±4.56 %, *n*=158). % Vul (vulvaless ratio). *$P <$ 0.001.
