# Supplementary material for: An In Vivo C. elegans Model System for Screening EGFR-Inhibiting Anti-Cancer Drugs
Source: PLoS One. 2012 Sep 5;7(9):e42441. doi: 10.1371/journal.pone.0042441 (PMC3434183; doi:10.1371/journal.pone.0042441)
Supplement: Figure S2 — Amino acid sequences of the LET-23::hEGFR-TK and LET-23::hEGFR transgene products. (PDF) [file pone.0042441.s002.pdf]

A

MRYPPSIGSILLIIPIFLTFFGNSNAQLWKRCVSPQDCLCSGTTNGISRYGTGNILEDLETMYRGCRRVYGNLEITWIEA
NEIKKWRESTNSTVDPKNEDSPLKSINFFDNLEEIRGSLIIYRANIQKISFPRLRVIYGDEVFHDNALYIHKNDKVHEVV
MRELRVIRNGSVTIQDNPKMCYIGDKIDWKELLYDPDVQKVETTNSHQHCYQNGKSMAKCHESCNDKCWGSGDNDCQRVY
RSVCPKSCSQCFYSNSTSSYECCDSACLGGCTGHGPKNCIACSKYELDGICIETCPSRKIFNHKTGRLVFNPDGRYQNGN
HCVKECPPELLIENDVCVRHCSDGHHYDATKDVRECEKCRSSSCPKICTVDGHLTNETLKNLEGCEQIDGHLIIEHAFTY
EQLKVLETVKIVSEYITIVQQNFYDLKFLKNLQIIEGRKLHNVRWALAIYQCDDLEELSLNSLKLIKTGAVLIMKNHRLC
YVSKIDWSSIITSKGKDNKPSLAIAENRDSKLCETEQRVCDKNCNKRGCWGKEPEDCLECKTWKSVGTCVEKCDTKGFLR
NQTSMKCERCSPECETCNGLGELDCLTCRHKTLYNSDFGNRMECVHDCPVSHFPTQKNVCEKCHPTCYDNGCTGPDSNLG
YGGCKQCKYAVKYENDTIFCLQSSGMNNVCVENDLPNYYISTYDTEGVIETHCEKCSISCKTCSSAGRNVVQNKCVCKHV
EYQPNPSERICMDQCPVNSFMVPDTNNTVCKKCHHECDQNYHCANGQSTGCQKCKNFTVFKGDIAQCVSECPKNLPFSNP
ANGECLDYDIASRQRKTRMVIIGSVLFGFAVMFLFILLVYW**RRRHIVRKRTLRRLLQERELVEPLTPSGEAPNQALLRIL**
KETEFKKIKVLGSGAFGTVYKGLWIPEGEKVKIPVAIKELREATSPKANKEILDEAYVMASVDNPHVCRLLGICLTSTVQ
LITQLMPFGCLLDYVREHKDNIGSQYLLNWCVQIAKGMNYLEDRRLVHRDLAARNVLVKTPQHVKITDFGLAKLLGAEEK
EYHAEGGKVPIKWMALESILHRIYTHQSDVWSYGVTVWELMTFGSKPYDGIPASEISSILEKGERLPQPPICTIDVYMIM
VKCWMIDADSRPKFRELIIEFSKMARDPQLFLENSNKISESDLSAEERFQTERIREMFDGNIDPQMYFDQGSLPSMPSSP
TSMATFTIPHGDLMNRMQSVNSSRYKTEPFDYGSTAQEDNSYLIPKTKEVQQSAVLYTAVTNEDGQTELSPSNGDYYNQP
NTPSSSSGYYNEPHLKTKKPETSEEAEAVQYENEEVSQKETCL

B

MRYPPSIGSILLIIPIFLTFFGNSNAQLWKRCVSPQDCLCSGTTNGISRYGTGNILEDLETMYRGCRRVYGNLEITWIEA
NEIKKWRESTNSTVDPKNEDSPLKSINFFDNLEEIRGSLIIYRANIQKISFPRLRVIYGDEVFHDNALYIHKNDKVHEVV
MRELRVIRNGSVTIQDNPKMCYIGDKIDWKELLYDPDVQKVETTNSHQHCYQNGKSMAKCHESCNDKCWGSGDNDCQRVY
RSVCPKSCSQCFYSNSTSSYECCDSACLGGCTGHGPKNCIACSKYELDGICIETCPSRKIFNHKTGRLVFNPDGRYQNGN
HCVKECPPELLIENDVCVRHCSDGHHYDATKDVRECEKCRSSSCPKICTVDGHLTNETLKNLEGCEQIDGHLIIEHAFTY
EQLKVLETVKIVSEYITIVQQNFYDLKFLKNLQIIEGRKLHNVRWALAIYQCDDLEELSLNSLKLIKTGAVLIMKNHRLC
YVSKIDWSSIITSKGKDNKPSLAIAENRDSKLCETEQRVCDKNCNKRGCWGKEPEDCLECKTWKSVGTCVEKCDTKGFLR
NQTSMKCERCSPECETCNGLGELDCLTCRHKTLYNSDFGNRMECVHDCPVSHFPTQKNVCEKCHPTCYDNGCTGPDSNLG
YGGCKQCKYAVKYENDTIFCLQSSGMNNVCVENDLPNYYISTYDTEGVIETHCEKCSISCKTCSSAGRNVVQNKCVCKHV
EYQPNPSERICMDQCPVNSFMVPDTNNTVCKKCHHECDQNYHCANGQSTGCQKCKNFTVFKGDIAQCVSECPKNLPFSNP
ANGECLDYDIASRQRKTRMVIIGSVLFGFAVMFLFILLVYW**RRRHIVRKRTLRRLLQERELVEPLTPSGEAPNQALLRIL**
KETEFKKIKVLGSGAFGTVYKGLWIPEGEKVKIPVAIKELREATSPKANKEILDEAYVMASVDNPHVCRLLGICLTSTVQ
LITQLMPFGCLLDYVREHKDNIGSQYLLNWCVQIAKGMNYLEDRRLVHRDLAARNVLVKTPQHVKITDFGLAKLLGAEEK
EYHAEGGKVPIKWMALESILHRIYTHQSDVWSYGVTVWELMTFGSKPYDGIPASEISSILEKGERLPQPPICTIDVYMIM
VKCWMIDADSRPKFRELIIEFSKMARDPQRYLVIQGDERMHLPSPTDSNFYRALMDEEDMDDVVDADEYLIPQQGFFSSP
STSRTPLLSSLSATSNNSTVACIDRNGLQSCPIKEDSFLQRYSSDPTGALTEDSIDDTFLPVPEYINQSVPKRPAGSVQN
PVYHNQPLNPAPSRDPHYQDPHSTAVGNPEYLNTVQPTCVNSTFDSPAHWAQKGSHQISLDNPDYQQDFFPKEAKPNGIF
KGSTAENAEYLRVAPQSSEFIGAKETCL

Figure S2. The amino acid sequences of the LET-23::hEGFR-TK (A) and LET-23::hEGFR (B) transgene products. The regular characters are LET-23 amino acids and the bolded characters are human EGFR amino acids. Each line includes 80 amino acids.
