# Supplementary material for: An In Vivo C. elegans Model System for Screening EGFR-Inhibiting Anti-Cancer Drugs
Source: PLoS One. 2012 Sep 5;7(9):e42441. doi: 10.1371/journal.pone.0042441 (PMC3434183; doi:10.1371/journal.pone.0042441)
Supplement: Figure S3 — Knock-down of Ras-MAPK and Wnt pathway genes in jgIs6 and jgIs25 by feeding RNAi. (PDF) [file pone.0042441.s003.pdf]

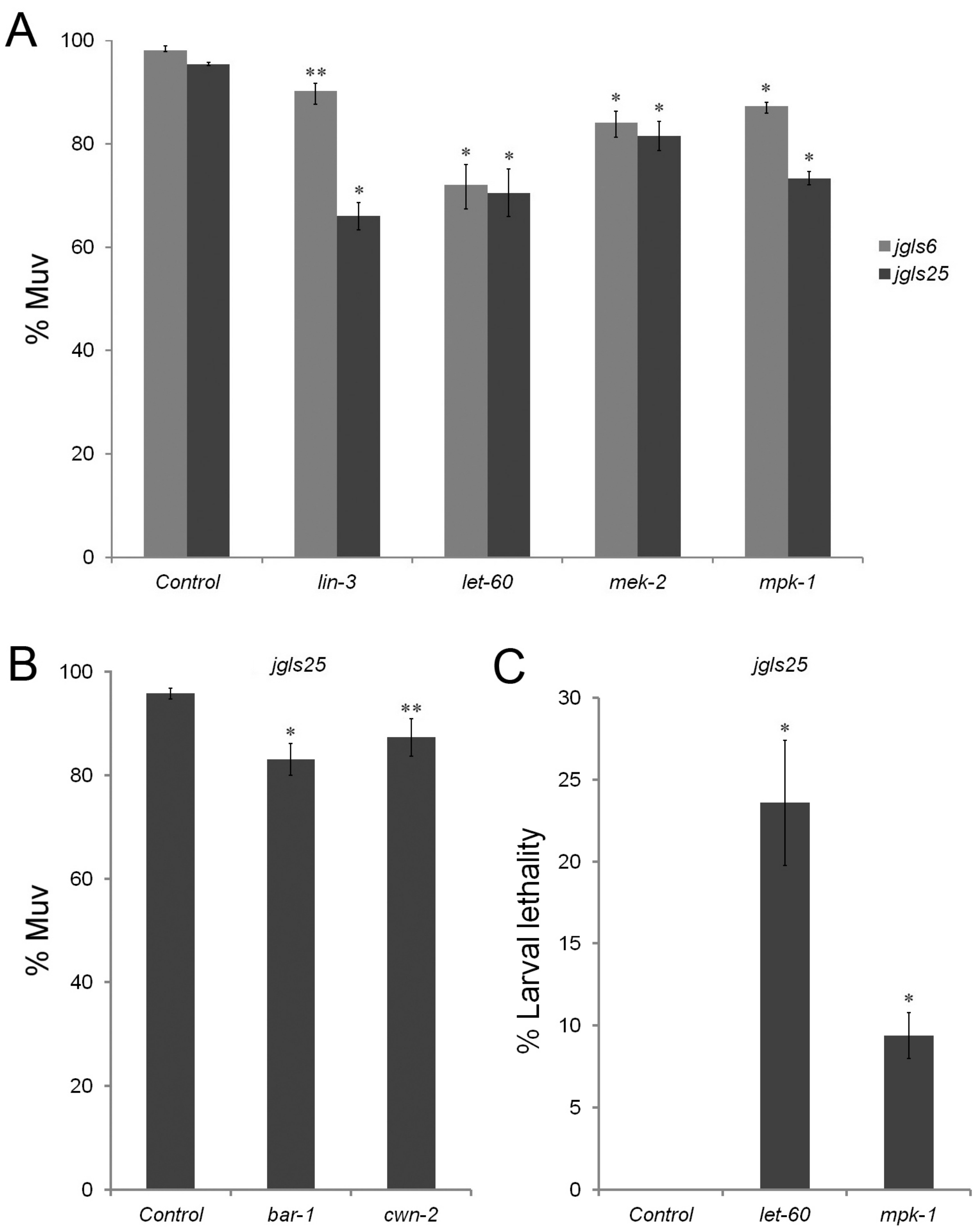


**Figure S3.** Knock-down of Ras-MAPK and Wnt pathway genes in *jgIs6* and *jgIs25* by feeding RNA*i*. (A) The Muv phenotype is suppressed by RNAi of EGFR downstream genes such as *let-60, mek-2* and *mpk-1*. Knock-down of the EGFR upstream gene *lin-3* also suppressed the Muv phenotype. (*n* = 247*,* 417, 299, 457, 262, 113, 565, 351, 522 and 626 from left along the X-axis). RNAi was started with synchronized L1 larvae, the same method used for drug treatment. Therefore, we observed very little larval lethality; *jgIs25* treated with control L4440 RNAi (0%) and *jgIs25* treated with *let-60* RNAi (0.6±0.31%). (B) The Muv phenotype of *jgIs25* is suppressed by RNAi of

the Wnt pathway genes. We tested two *Wnt* genes, *bar-1* (a β-catenin) and *cwn-2* (a Wnt ligand). Knock-down of the Wnt pathway suppressed the Muv phenotype, but its effect is lesser than knock-down of the EGFR pathway. (*n*= 261*,* 450, and 602 from left along the X-axis). (C) Larval lethality of RNAi fed to *jgIs25* L4 larvae. To confirm the RNAi effect of lethal EGFR downstream genes, we performed *let-60* or *mpk-1* RNAi on *jgIs25* L4 larvae, and counted the numbers of lethal F1 progeny during larval development. Laval lethality was significantly increased by *let-60* or *mpk-1* RNAi. (*n*= 273*,* 419, and 254 from left along the X-axis). All feeding RNAi clones from the Ahringer feeding RNAi library are kindly provided by Dr. Lee (SNU, Seoul, Korea). X-axis (RNAi of each gene). Control (L4440 feeding). *$P < 0.001$ and **$P < 0.05$.
