# Supplementary material for: An In Vivo C. elegans Model System for Screening EGFR-Inhibiting Anti-Cancer Drugs
Source: PLoS One. 2012 Sep 5;7(9):e42441. doi: 10.1371/journal.pone.0042441 (PMC3434183; doi:10.1371/journal.pone.0042441)
Supplement: Figure S4 — Expression of epithelial junction proteins in the jgIs6 transgenic worm which expresses LET-23::hEGFR-TK[L858R]. (PDF) [file pone.0042441.s004.pdf]

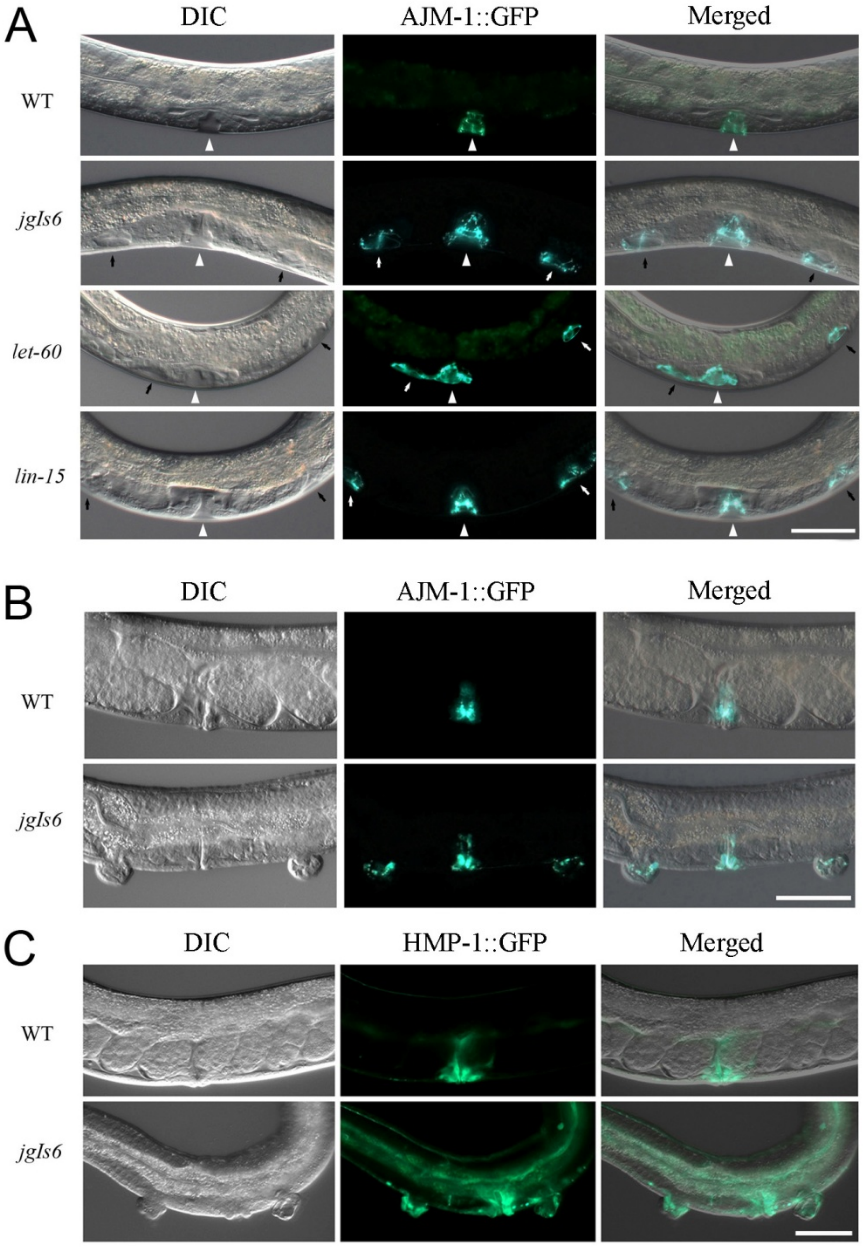

Figure S4. Expression of epithelial junction proteins in the *jgIs6* transgenic strain which expresses LET-23::hEGFR-TK[L858R]. (A) AJM-1::GFP expression at the L4 stage of wild type, Muv mutants and *jgIs6*. Arrowheads indicate normal vulval invaginations and small arrows indicate pseudovulval invaginations. (B) AJM-1::GFP expression at the adult stage of the wild-type strain and *jgIs6*. (C) HMP-1::GFP expression in the wild-type strain and *jgIs6*. Scale bars, 50 µm.
