# Supplementary material for: An In Vivo C. elegans Model System for Screening EGFR-Inhibiting Anti-Cancer Drugs
Source: PLoS One. 2012 Sep 5;7(9):e42441. doi: 10.1371/journal.pone.0042441 (PMC3434183; doi:10.1371/journal.pone.0042441)
Supplement: Figure S5 — Adult ratios of wild type and two integrated strains over time. (PDF) [file pone.0042441.s005.pdf]

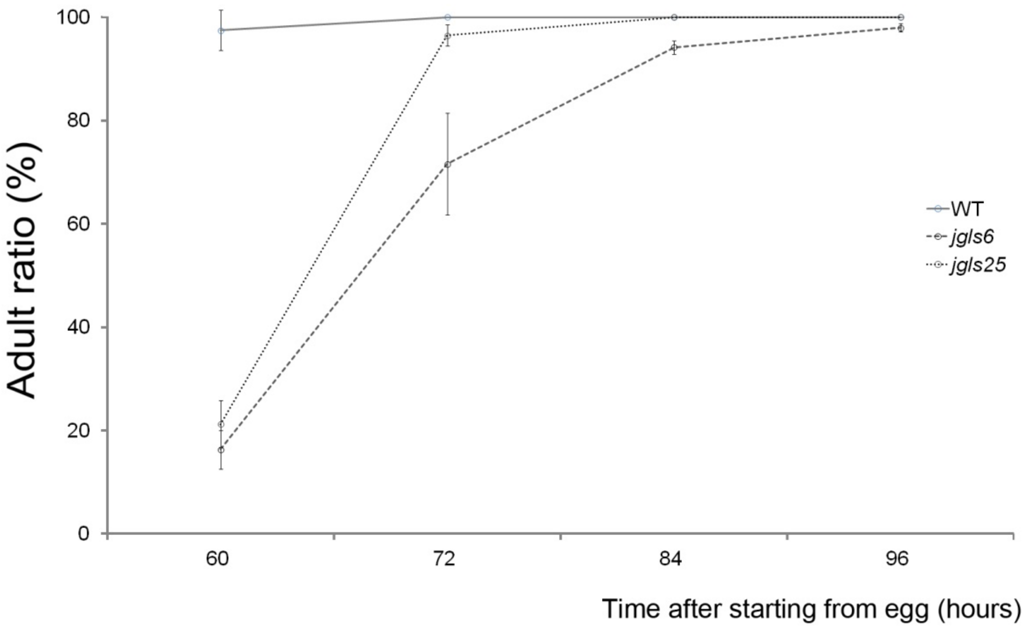

Figure S5. Adult ratios of the wild type and two integrated strains across time. *jgIs6* showed the slowest growth phenotype. At 60 hours from eggs placed on the plate, most wild type are adult (97.4±3.92%), but the two integrated strains showed low adult ratios (16.3±3.72% for *jgIs6* and 21.2±4.59% for *jgIs25*). Only *jgIs6* showed the larval arrested phenotype (1.8±0.78%). The numbers of worms counted are 216 (WT), 283 (*jgIs6*) and 267 (*jgIs25*). We tested 3 plates for each strain and calculated the standard deviation. WT (wild-type N2).
