# Supplementary material for: An In Vivo C. elegans Model System for Screening EGFR-Inhibiting Anti-Cancer Drugs
Source: PLoS One. 2012 Sep 5;7(9):e42441. doi: 10.1371/journal.pone.0042441 (PMC3434183; doi:10.1371/journal.pone.0042441)
Supplement: Figure S6 — Seven chemicals found to inhibit the Muv phenotype of jgIs6 in another screen are similar in structure to gefitinib. (PDF) [file pone.0042441.s006.pdf]

Chemicals	% inhibition	Common backbone
U-000572-B04	87.30%	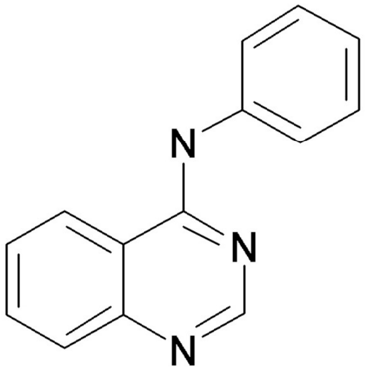
U-000506-G06	93.70%	
U-000506-E11	76.20%	
U-000512-H08	91.40%	
U-000511-E06	85.90%	
U-000511-G07	95.90%	
U-000511-D05	94.60%	

Figure S6. Seven chemicals found to inhibit the Muv phenotype of *jgIs6* in another screen are similar in structure to gefitinib. 7,680 chemicals which are unknown for their target and pathway were used in this screen. This chemical library was provided by Korea Chemical Bank.
